# AQbD-Based UPLC-ELSD Method for Quantifying Medium Chain Triglycerides in Labrafac™ WL 1349 for Nanoemulsion Applications

**DOI:** 10.3390/molecules30030486

**Published:** 2025-01-22

**Authors:** Alessio Gaggero, Viktoria Marko, Dalibor Jeremic, Carolin Tetyczka, Philippe Caisse, Jesús Alberto Afonso Urich

**Affiliations:** 1 Research Center Pharmaceutical Engineering GmbH, 8010 Graz, Austria; alessio.gaggero@rcpe.at (A.G.); viktoria.marko@rcpe.at (V.M.); carolin.tetyczka@rcpe.at (C.T.); 2 Department of Health Studies—Biomedical Science, FH JOANNEUM, 8020 Graz, Austria; dalibor.jeremic@fh-joanneum.at; 3 Gattefossé SAS, 69804 Saint-Priest, France; pcaisse@gattefosse.com; 4 Institute of Process and Particle Engineering, Graz University of Technology, 8010 Graz, Austria

**Keywords:** medium chain triglycerides, evaporative light scattering detection, analytical quality by design, nanoemulsions

## Abstract

In response to recent regulatory guidelines, including ICH (International Council for Harmonisation) Q2 (R2) and Q14, we developed a UPLC-ELSD method to quantify Medium-Chain Triglycerides (MCTs) in Labrafac™ WL 1349 for nanoemulsion applications. This procedure, crafted using Analytical Quality by Design (AQbD) principles, addresses not only the validation of the methodology but also the lifecycle management challenges associated with the analysis of lipid-based excipients. Key parameters such as mobile phase composition, organic modifier, column type, flow rate, diluent, and column temperature were optimized to meet regulatory standards and ensure robustness in MCT quantification. Optimal conditions were achieved with a Waters Acquity HSS T3 (100 × 2.1 mm i.d., 1.8 μm) column at 33 °C, using a mixture of methanol (97.5%) and water (2.5%) containing 0.4% of formic acid at a flow rate of 0.41 mL/min. The method demonstrated an excellent fit on a cubic modelization for MCTs over a broad range of concentrations. Forced degradation studies, including hydrolytic (acidic and basic), oxidative, and thermal stress, confirmed the method’s suitability for possible stability scenarios. This validated UPLC method was successfully applied to quantitative analyses of bulk and formulation prototype samples containing MCTs. This AQbD-driven method enhances not only knowledge but also regulatory-compliant and cost-effective excipient control.

## 1. Introduction

Labrafac^TM^ WL 1349 (Labrafac) is a pharmaceutical excipient composed of Medium-Chain Triglycerides (MCTs), primarily derived from caprylic (C8) and capric (C10) fatty acids. It is presented in the form of an oily liquid vehicle and is known for its excellent solubilizing properties. According to the Lipid Formulation Classification System (LFCS), Labrafac can widely contribute to formulating lipid-based drug delivery systems (LBDDS). This includes type I, type II, and type III, which are defined as oils, self-emulsifying drug delivery systems (SEDDS), and self-microemulsifying drug delivery systems (SMEDDS) [1]. Moreover, Labrafac can also play a role in formulating other kinds of lipid nanoparticles, like Nanostructured Lipid Carriers (NLC), composed of solid and liquid lipids [2]. Among these advanced formulation strategies, promising approaches like microemulsions and nanoemulsions (NEs) can be found.

Systems like NEs are particularly beneficial for enhancing the solubility and bioavailability of poorly water-soluble lipophilic drugs [3], as they are developed thanks to the addition of surfactants, capable of reducing the interfacial tension between the oil and the water phase [4]. A thermodynamically stable NE is the result of applying shear forces to the combined phases, where it is critical to select an optimal mixture of low and high hydrophilic-lipophilic balance (HLB) surfactants and lipids to guarantee the shaping of a stable NE [5,6]. Oily vehicles like Labrafac are regularly utilized in producing NEs, where their characterization consists of evaluating parameters such as droplet size, structure, and kinetical behavior. The droplet size is a significant variable that influences the stability of NEs, considering that they mostly undergo the Ostwald ripening effect, which may considerably vary the droplet size distribution and lead to emulsion polydispersity [7]. A non-homogeneous droplet size pattern inevitably results in the NE’s unpredictable stability behavior and consequent uncertain therapeutic effects.

A large-scale production line for NEs comprises multiple steps, and the methodologies can vary using high and low-energy preparation methods [8]. Given the inherited complexity of these processes, it would be considerably ambitious nowadays to encompass the stability topic just by defining droplet size interval limits at pre-defined storage conditions. The effect that the excipients’ concentration changes across time could provide on the formulation’s stability should also be addressed. The stability discussion often refers to the stability of the Active Pharmaceutical Ingredient (API) itself (e.g., regular control of related substances) in light of the drug monographs proposed by the leading regulatory framework: in the case of the NEs, it’s fundamental to provide a strict control on the excipients as well, whose concentration variations might potentially lead to unstable, and thus unsafe and inefficient pharmaceuticals [9]. Although NEs are usually reputed as stable, thanks to the lower probability of creaming, flocculation, and coalescence appearing, the potential effect of the inert ingredients blend on their stability cannot be excluded [7].

Routine control analysis of lipids has already been extensively reported in the literature [10,11,12,13], where Ultra High-Performance Liquid Chromatography (UPLC) utilizing a reversed-phase (RP) approach represents the most efficient solution time-wise and cost-wise [14]. However, the European Pharmacopeia 11.0 still proposes a method for the detection of MCT’s fatty acids composition via Gas Chromatography (GC) in the respective monograph [15]. The advantage of using HPLC-RP compared to GC lies in the possibility of avoiding the derivatization step necessary for analyzing non-volatile lipids [16]. Regarding detection, MCTs, like many triglycerides, are not easily detectable via UV mode due to the lack of chromophore groups [17]. Additionally, the absence of readily ionizable functional groups complicates MS detection. Therefore, alternative determination strategies need to be adopted to quantify lipids successfully: among these, Evaporative Light Scattering Detection (ELSD) provides excellent sensitivity and separation of lipids less volatile than the mobile phase [12]. The detector works by nebulizing the column’s effluents into a fine aerosol mist, then moving through a heated drift tube, where the mobile phase evaporates. Hence, the remaining non-volatile residues are detected based on the amount of light scattered and proportional to the amount of signal generated [18].

**Figure 1:**
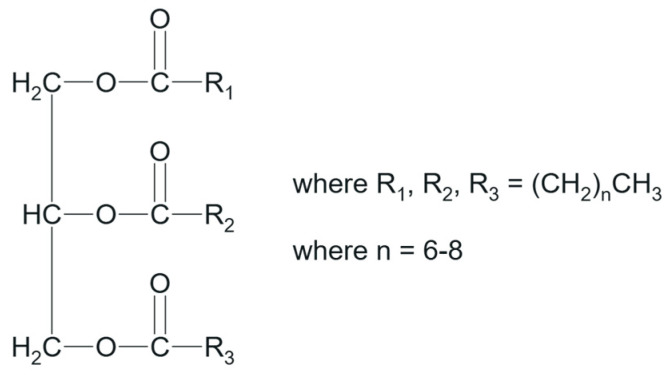
Labrafac structure, including possible combinations of triglyceride fatty acid mixtures.

Nowadays, ELSD efficiently translates the necessity of having a “universal detection technique”, where the main requirement for the analyte is to be at least semi-volatile [19]. Granted the detector’s capability of identifying many different species, the method development for the quantification of MCTs contained in Labrafac should aim to sufficiently separate the different esters mixtures of caprylic (C8) and capric (C10) acids, and thus provide knowledge around the conformation and stability of the NE. Effectively, the procedure needs to target four distinct combinations of C8 and C10 [20]. An exemplary structure of Labrafac is given below in Figure 1.

Interestingly, the MCTs USP monograph does not include strict limits for the quantification of Labrafac triglyceride fatty acids, whose specification ranges lie between 50.0 and 80.0% for C8 and between 20.0 and 50.0% for C10, further justifying the necessity of regular control [15,20]. It is not excluded that the methodology could detect the remaining fatty acid derivatives from caproic, lauric, and myristic acid, as well as forms of USP’s Mono and Diglycerides [21].

The analytical constraints presented by Labrafac’s complex matrix underline the exigency of meticulous method development and validation: in this scenario, the Analytical Quality by Design (AQbD) principles offer a systematic approach to design a robust method with the desired degree of performance [22,23]. AQbD is an extension of the Quality by Design (QbD) framework defined by the European Medicines Agency (EMA), which encourages employing these principles for developing methods and manufacturing drugs with expected high-quality standards, following the ICH Q8 guideline [24,25]. Validating an analytical procedure with the AQbD approach strongly increases its reliability and consistency, providing a robust structure for critical studies like stability [26]. The first step of the AQbD workflow includes defining the Analytical Target Profile (ATP), which comprises Critical Method Attributes (CMeAs) and performance characteristics, pivotal aspects to ensure that the method fits its intended target [27]. The ATP defines the level of quality planned for the procedure, and results obtained from the chromatographic sequences should meet reasonable standards of accuracy and precision, defined by the ICH Q2 (R2) guideline [28]. The validation followed the same compendium, involving a detailed evaluation of the response, limit of detection (LOD), limit of quantification (LOQ), specificity, accuracy, precision, and robustness. As recommended in the last update of the ICH Q14 guideline, robustness and risk assessment data were acquired by using Design of Experiments (DoE), a fundamental tool to assess the relationship between CMeAs and Critical Method Parameters (CMePs), pre-selected factors that could potentially influence Labrafac’s determination [29,30]. Additionally, DoE reduces the number of runs needed to achieve the desired chromatographic conditions to fit the ATP, while it was reported to reduce out-of-trend (OOT) and out-of-specification (OOS) results [29,31]. The AQbD workflow proceeds with statistically defining the Method Operable Design Region (MODR), which is the area where the responses provided by the analytical results can be predicted within a specific range, and the method can be used [32]. Once the analytical procedure is established and validated, its lifecycle is monitored to guarantee the results’ reproducibility across time [33].

Detecting lipids constitutes a challenge in terms of retention in reversed-phase mode: the choice of mobile phase and different chromatographic parameters are critical factors to consider, and this work aims to present a robust analytical methodology capable of achieving optimal retention of the target Labrafac peaks by using solid statistical tools such as DoE and systematic approaches like the AQbD principles while defining strict boundaries for its routine use.

## 2. Results and Discussion

### 2.1. Method Development by AQbD Principles

**Figure 2:**
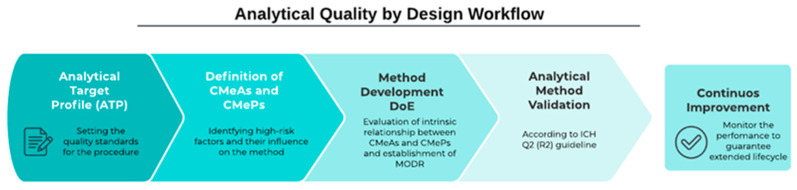
Analytical Quality by Design workflow for the development of the Labrafac procedure.

The AQbD workflow comprises multiple steps that align with the recently updated USP <1220> chapter and ICH Q14 revisions [30,33]. Correct execution of the AQbD process guarantees the extension of the analytical procedure lifecycle, a fundamental characteristic that confirms the method’s robustness and reliability [34]. A summary of the steps involved in the AQbD development is provided in Figure 2.

The ATP definition is the initial stage of the AQbD process, which sets the standard performance requirements the method should meet. As previously mentioned, Labrafac is a mixture of multiple triglyceride species, and this procedure could detect four different peaks, recognized as tricaprylin, 1,2-caprate-3-caprylate, 1,2-caprylate-3-caprate, and tricaprin. The peak’s identity was additionally confirmed by injecting the standards of the abovementioned components. Thus, the method was asked to maintain reproducibility regarding retention time (RT), sensitivity, accuracy, and precision throughout the development and validation progress. The ELSD response is highly subjected to variations in sensitivity depending on the pre-selection of the detector’s parameters, such as drift tube temperature, and significant increases may lead to the disappearance of some of the peaks due to evaporation. For this reason, the final method should show all the initially detected components [35]. The chosen requirements for this analytical methodology are summarized in the ATP shown in Table 1.

**Figure 3:**
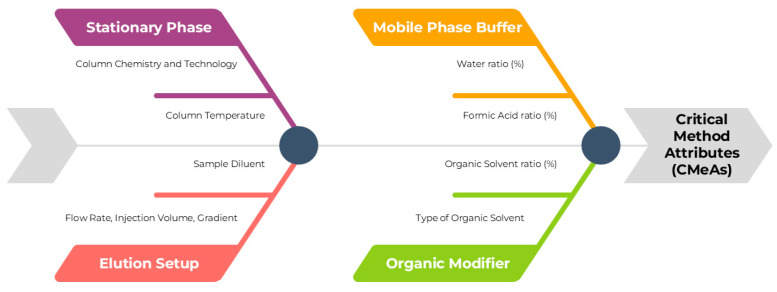
Ishikawa Fishbone Diagram for the assessment of CMeAs and CMePs.

Triglycerides are not suitable candidates for conventional UV detection due to the lack of chromophore groups, thus a different technique should be explored to develop the method. In this scenario, lipids are usually detected via either GC or ELSD. Compared to GC analysis, ELSD methodologies represent a cheaper and more time-effective alternative for characterizing lipids, while cutting out the derivatization step, offering a broader detection range, and fastening analysis time. ELSD constitutes a significant improvement in the field while using traditional reverse-phase chromatography, and this publication aims to present an alternative methodology to the official procedure stated in the Ph. Eu. monograph [15,37]. Critical Method Parameters (CMePs) have been selected to facilitate the identification of the Critical Method Attributes (CMeAs), and a quality risk management (QRM) tool like the Ishikawa fishbone diagram was employed to assess the intrinsic relationship (Figure 3).

Given the inherited complexity of Labrafac’s structure, a series of eight high-risk factors were evaluated, and the existing relationship of significance was assessed with a first DoE meant to screen the method’s chromatographic features. A set of four C18 columns with different hydrophobic indices was screened to reach optimal retention, in combination with injection volume, flow rate, sample diluent, column temperature, organic modifier, percentage of aqueous phase, and percentage of formic acid (FA) in the mobile phase. Labrafac is a very hydrophobic compound, which means that canonic mobile phases, including a higher water ratio used in standard reversed-phase chromatography, cannot provide sufficient retention, leading to unsatisfactory selectivity and resolution [38]. A considerable ratio of an organic phase was utilized as the primary eluent, with the addition of small quantities of water and FA, suitable for ELSD procedures to provide optimal separation while controlling the mobile phase pH [39]. As for the solubility, preliminary trials proved that Labrafac visually dissolved in the selected organic solvents.

This first screening focused attention on critical chromatographic parameters while developing analytical procedures to establish a baseline for our method (Table 2). Standardizing these parameters is crucial to obtaining a methodology that fits the intended purpose of the ATP. Pivotal factors coming from the detector’s configuration, like drift tube temperature, gas pressure, and nebulizer power, have an important influence on the sensitivity and reproducibility of the results [40], and they were evaluated separately in a subsequent DoE utilized to optimize and ensure the method’s robustness during the validation process. Afterward, the procedure was validated following the latest ICH Q2 (R2) guideline (Validation of Analytical Procedures), meeting defined standards of accuracy and precision [28].

### 2.2. Statistical Evaluation of the Development DoE and Definition of MODR

A comprehensive DoE approach was implemented, utilizing an I-optimal response surface design with coordinate exchange to thoroughly explore the multidimensional parameter space based on the variable’s number of identified peaks, capacity factor, peak height, resolution, and peak tailing (Table 1). This sophisticated experimental design strategy was chosen to simultaneously optimize multiple critical method parameters while investigating potential quadratic effects and parameter interactions. This systematic approach aimed to establish a reliable analytical method capable of consistent triglyceride separation.

The execution of the initial experimental design revealed several significant challenges that required adaptive solutions. The primary obstacle emerged when certain experimental conditions failed to resolve all four expected peaks of the triglyceride mixture. This variable peak count necessitated the exclusion of multiple experimental runs, consequently leading to the aliasing of quadratic terms in the statistical model. Despite this setback, the experimental design maintained sufficient power to identify critical method parameters and their 2-way interactions.

The DoE approach successfully identified robust operating conditions, described in Table 3. The developed method’s reproducibility was confirmed through a series of six confirmation runs under the optimized conditions. Results fell within the 95% prediction interval for all critical method attributes related to chromatography. This statistical validation demonstrated that the method successfully maintained chromatographic performance.

**Figure 4:**
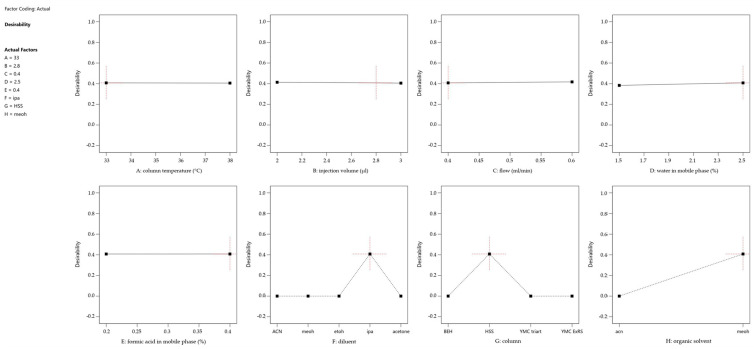
Prediction Profiler showing the impact of each factor setting on the overall desirability. The red cross indicates the final method setting. Factor coding: A = column temperature (°C), B = injection volume (µl), C = flow (ml/min), D = water amount in mobile phase (%), E = formic acid amount in mobile phase (%), F = diluent, G = column, H = organic solvent.

**Figure 5:**
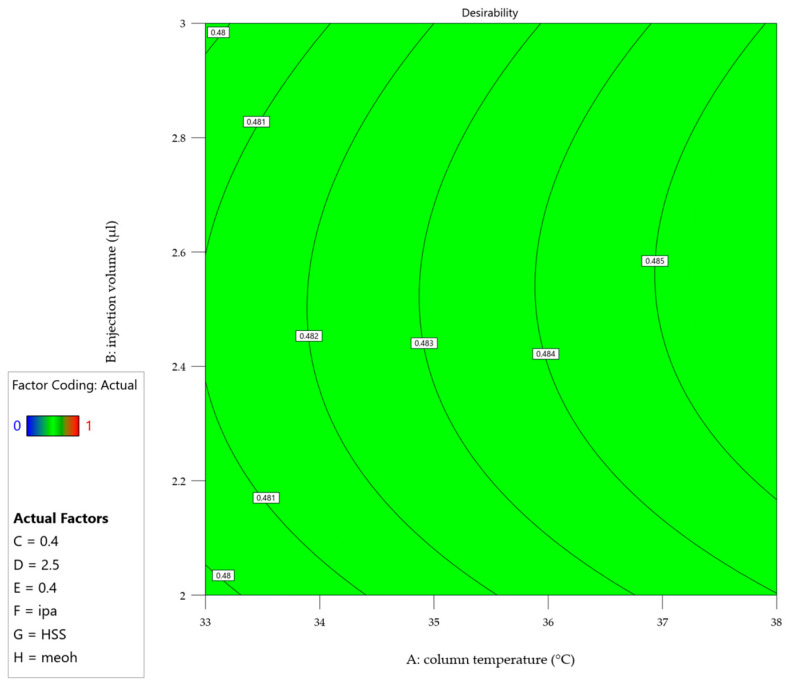
Contour plot of the desirability for the final factor settings according to the legend. Factor coding: A = column temperature (°C), B = injection volume (µl), C = flow (ml/min), D = water amount in mobile phase (%), E = formic acid amount in mobile phase (%), F = diluent, G = column, H = organic solvent.

To address the challenge of optimizing multiple responses simultaneously, a desirability function was incorporated into the prediction profiler (Figure 4). This function combines the individual response predictions into a single desirability index, ranging from 0 (undesirable) to 1 (highly desirable), allowing for the identification of factor combinations that maximize overall performance across all responses. Figure 5 shows the desirability across a surface of factors A (Column Temperature) and B (Injection Volume) and fixed settings for the other factors, shown in the legend. 

Both figures revealed several key findings: the chromatographic parameters, including column temperature, injection volume, and flow rate, demonstrated remarkable stability across their studied ranges. A slight positive correlation was observed between the percentage of the aqueous phase and method performance, while formic acid concentration had minimal impact within the studied range. Additionally, optimal choices for categorical factors—such as column type, sample diluent, and organic modifier—were identified, enhancing the method’s overall performance. Furthermore, the optimization process highlighted an interdependence between column temperature and flow rate, with their selection supported by both theoretical and practical considerations. A lower temperature reduces the risk of non-compliant resolution and potential thermal stress on the analyte [41]. Similarly, a low flow rate enhances nebulization efficiency, allows complete solvent evaporation, which is proportional to the signal and reduces the likelihood of incomplete droplet formation [42]. Additionally, a lower flow rate provides, in principle, better resolution and lower overall pressure to be handled by the chromatographic system [43]. The DoE evaluation results are located in the Supplementary Material (Table S1).

### 2.3. Analytical Method Validation

Granted the optimal outcomes designated by the screening DoE, the final conditions of the analytical method were assessed (Table 3), and the same parameters were utilized for the validation. It’s important to underline that the calculations for the validation are made on the total amount of MCTs in Labrafac, considering every single peak amount together, including calibration.

**Figure 6:**
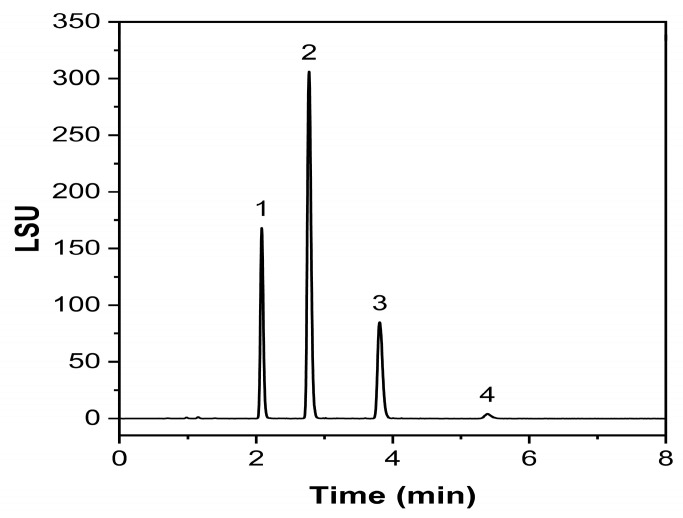
Exemplary chromatogram of a Labrafac injection. (1) tricaprylin, (2) 1,2-caprate-3-caprylate, (3) 1,2-caprylate-3-caprate, (4) tricaprin.

An exemplary chromatogram obtained with this validated methodology is given in Figure 6, where complete separation could be achieved in 8 min. 

#### 2.3.1. Specificity and Forced Degradation Studies

**Figure 7:**
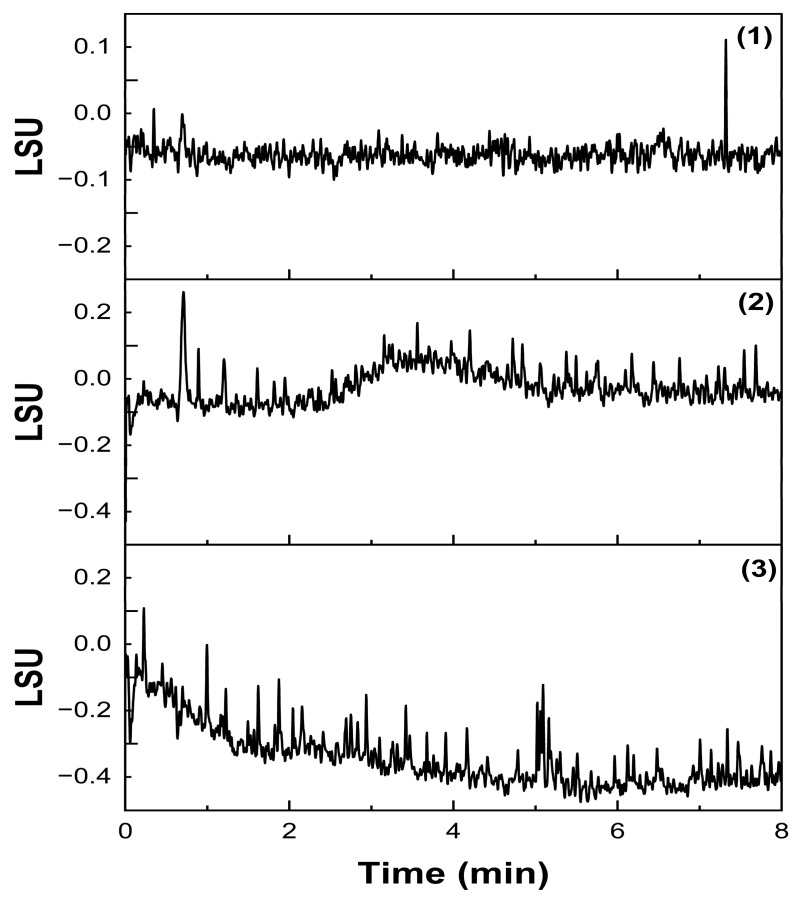
Chromatograms of specificity studies. (1) mobile phase injection; (2) blank injection; (3) nanoemulsion matrix injection.

Single injections of mobile phase, blank, and nanoemulsion matrix components exhibited no interference with the established Labrafac peaks, proving that the method is selective and specific for the compound of interest. The chromatograms are presented in Figure 7.

**Figure 8:**
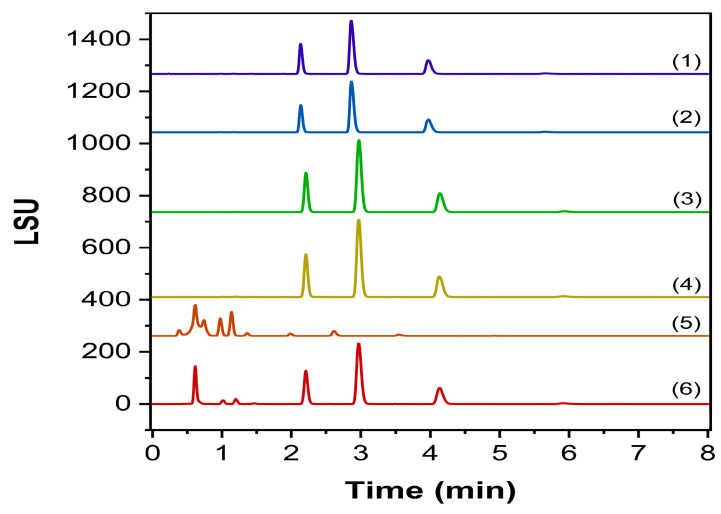
Chromatogram of forced degradation studies on Labrafac. (1) control sample, (2) RapidOxy stress, (3) thermal stress, (4) oxidative stress, (5) basic stress, (6) acidic stress.

The forced degradation study was conducted in triplicate, addressing possible co-elutions that might occur with our peaks of interest and evaluating the behavior of the existing Labrafac species. The chromatograms are displayed in Figure 8, as well as the results in Table 4.

**Figure 9:**
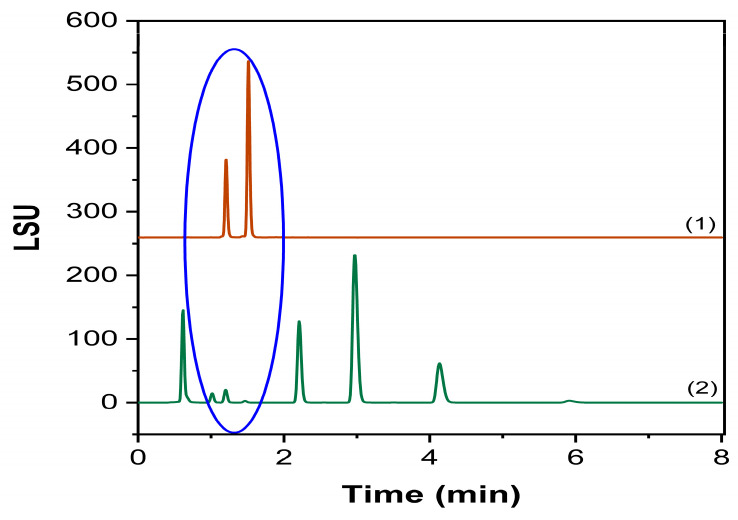
Comparison of Mono- and Diglycerides chromatograms. (1) Mono- and Diglycerides standard injection, (2) acidic stress condition. The blue circle identifies the same RT region.

The current regulatory framework encourages the implementation of forced degradation studies as a crucial part of the validation process, where the substance is accordingly subjected to different stress conditions, and the response is investigated [28]. Effectively, data obtained through these studies enforce the reliability of the analytical method, proving its specificity and suitability for the determined ATP. The developed procedure proved specificity regardless of the applied degrading conditions, without affecting the identification and quantification of the targeted Labrafac components. Although lipids are generally considered very prone to oxidation, Labrafac has been demonstrated to be resilient against RapidOxy and H_2_O_2_ stressing programs, confirming its key role in the stability of NEs. The acidic condition confirmed a hydrolysis pattern, promoting the appearance of two different products before the elution of tricaprylin, probably attributable to Mono- and Diglycerides. Following confirmatory runs evidenced the co-elution at the same RT of Mono- and Diglycerides [21], extending the methodology’s identification capability (Figure 9). Conversely, the alkaline condition thoroughly impacted the chromatography, greatly reducing the sensitivity of the Labrafac peaks but still within the acceptance criteria set by the ATP.

#### 2.3.2. Response, LOD, and LOQ

**Figure 10:**
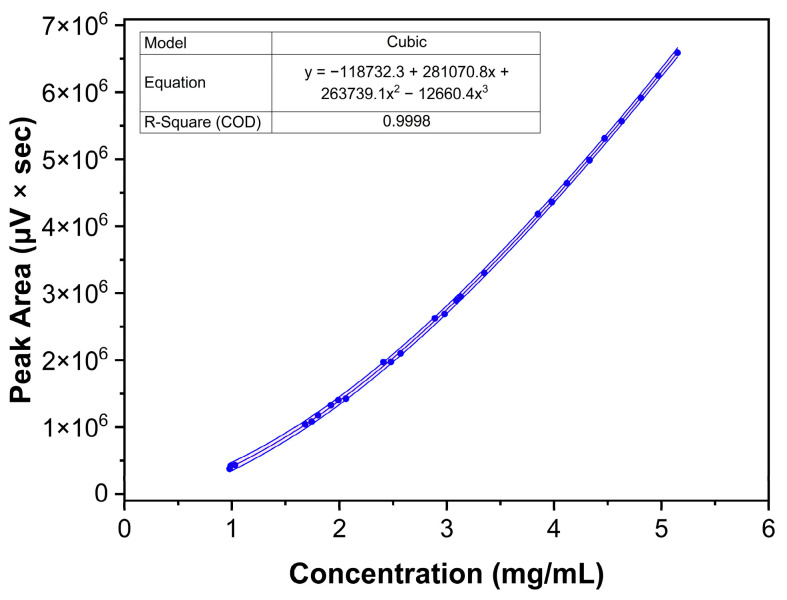
Combined regression data showing the model, the resulting equation, R^2^, and the 95% confidence intervals.

**Figure 11:**
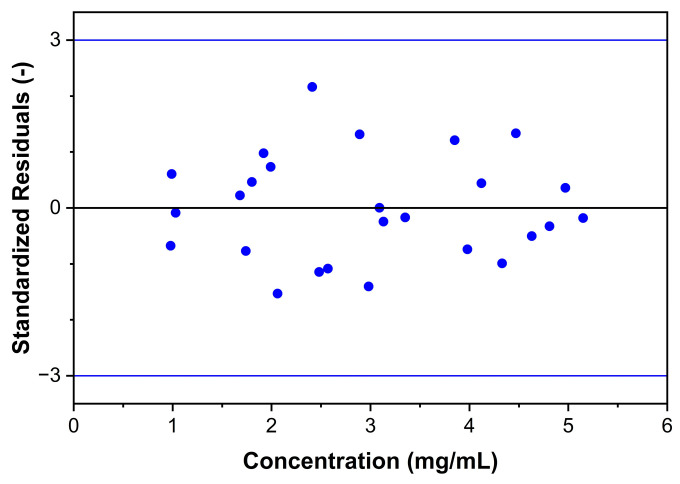
Standardized residuals for combined regression data.

Nine solutions appropriately distributed in the method’s working range were prepared in triplicate by weighing independent amounts of Labrafac and dissolving them in the chosen diluent. The method proved a non-linear response with a 3rd-order cubic fit in the range of 1.0 and 5.0 mg/mL and a correlation coefficient R^2^ = 0.9998, and the combined response plots are shown in Figure 10. Furthermore, the analysis of standardized residuals against predicted values from the regression model did not present apparent outliers or points of influence, as shown in Figure 11. The normal distribution of the standardized residuals was verified using the Shapiro-Wilk test (*p* = 0.821) [44].

LOD and LOQ were established from the estimated USP signal-to-noise ratios of prepared solutions at the lowest concentration (1.0 mg/mL), and the results are shown in Table 5.

#### 2.3.3. Accuracy and Precision (Repeatability and Intermediate Precision)

The accuracy was established based on mean recovery from a minimum of nine determinations distributed on three concentration levels (70, 100, and 130%) of the label/declared content claim, equal to 1.75, 2.5, and 3.25 mg/mL, respectively. Results proved that the method is accurate, displaying a recovery of 100 ± 2% (Table 6).

The developed method was demonstrated as precise and reproducible, and the RSD for each measurement was found to be below 2%. No statistical difference between the two analysts was ascertained, thus the two sets of samples were proven identical within the 95% confidence interval, with a *p* > 0.05 (Table 7).

#### 2.3.4. Robustness

Considering that the continuous factors showed little to no effect in the previous model, we conducted a robustness experiment for the settings of the ELSD itself. A response surface D-optimal design with 35 runs was executed. The factors investigated were the temperature of the drift tube (25–35 °C), the nebulizer gas rate (15–25%), and the gas pressure (35–45 psi). The same responses were evaluated as in the initial DoE.

The data were analyzed using a Type III ANOVA. No significant effects were detected at α = 0.05, except for the tailing of the tricaprylin peak, which showed a significant effect for the drift tube temperature (*p* = 0.015). While the calculated effect size (partial η^2^ = 0.26) of the drift tube setting on the tricaprylin tailing suggests a large effect, the actual tailing values varied only between 1.1 and 1.2, which is well within below limits for chromatographic methods, and therefore has no practical meaning. The statistical evaluation details of the robustness are shown in the Supplementary Material (Tables S2–S16).

#### 2.3.5. Nanopharmaceuticals Applications

**Figure 12:**
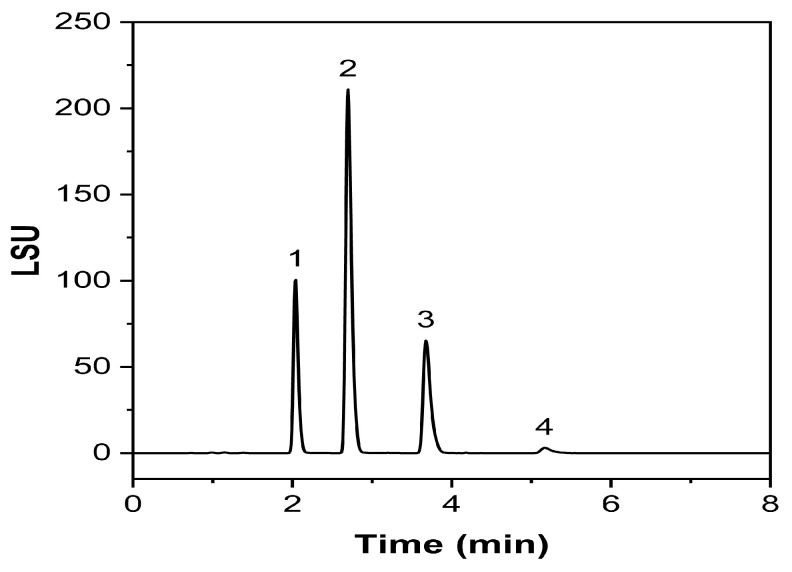
Exemplary chromatogram of the tested nanoemulsion prototype.

Due to their unique properties, lipid-based nanopharmaceuticals, including NEs and NLC, have gained increasing importance in various fields, especially in the pharmaceutical, cosmetics, and food industries. These nano-sized formulations with typically 20 to 200 nm droplet sizes offer improved stability, solubility, and, therefore, bioavailability of poorly soluble drugs. For many years, NEs excipients such as lipids and surfactants were considered inert substances. However, it is undeniable that certain excipients can have known or unknown interactions between excipients and APIs, other inactive ingredients, or a container closure system (CCS). Thus, it is essential to establish characterization methods for the excipients used in the early stages of the development of new formulations, especially for nano-drug delivery systems [45]. Consequently, the developed method was used to investigate an NE produced via impingement jet mixing. The results demonstrated (Figure 12) that the method can be used for Labrafac routine analysis in future studies.

#### 2.3.6. Analytical Procedure Lifecycle

Variability is inherent in routine analytical methods, impacting consistency in their performance [45]. Several foundational control strategies can be employed to maintain compliance with the Acceptance Test Procedure. Establishing specific control ranges for essential parameters—such as flow rate, pH, column temperature, and organic modifier concentration—helps create operational boundaries that optimize the method’s reliability. When these parameters exceed their limits, they signal potential issues, allowing timely adjustments that uphold method stability [46,47]. Ensuring consistency in reagent sources is another critical factor. By using the same manufacturers for primary reagents, particularly ammonium acetate buffer and chromatographic columns, the method benefits from consistent quality and composition, thereby reducing variability in results [47].

In addition, control charts are valuable for tracking daily chromatographic performance indicators, such as peak shape, retention time, and signal intensity. These charts visually capture trends over time, enabling early detection of unexpected changes or deviations. Together, these control strategies support the analytical method’s robustness, allowing for proactive monitoring and swift corrective action to ensure precise and reliable performance [33,47].

Future monitoring and data collection will further refine method robustness, contributing additional insights into long-term performance and supporting continuous improvement through control chart analyses.

## 3. Materials and Methods

### 3.1. Reagents and Consumables

The solvents used for the mobile phases, like Acetonitrile and Methanol (HPLC gradient), were purchased from M&B Stricker Laborfachhandel GbR (Bernried am Starnberger See, Germany). 2-Propanol used as a diluent and formic acid ≥ 98% used for pH modifications were purchased from Carl Roth (Karlsruhe, Germany). More chemicals used for analysis and forced degradation studies, like Ethanol absolute (HPLC-grade), 30% *w*/*v* Hydrogen Peroxide, Acetone (HPLC-grade), Sodium hydroxide pellets, and Hydrochloric Acid were acquired from Sigma-Aldrich GmbH (Vienna, Austria). The purification equipment Triton UV from Neptec (Elbtal, Germany) obtained the purified water for all analyses. All sample solutions were filtered before injecting into the chromatographic systems using nylon syringe filters (0.22 μm) from YETI Merz Brothers GmbH (Haid, Austria).

### 3.2. Standards, Samples and Excipients

The standards for identification of tricaprylin ≥ 97% and tricaprin ≥ 98% were acquired by Sigma Aldrich GmbH (Vienna, Austria), meanwhile, 1,2-caprate-3-caprylate and 1,2-caprylate-3-caprate were purchased from Larodan (Solna, Sweden). The Labrafac lipophile^TM^ WL 1349 used for the samples was kindly provided by Gattefossè SAS (Saint-Priest, France). Geleol Mono- and Diglycerides for identification and specificity were provided by Gattefossé SAS as well, while Tween^®^ 80 was acquired from Sigma-Aldrich GmbH (Vienna, Austria).

### 3.3. Equipment

A Reversed Phase Ultra Performance Liquid Chromatograph (UPLC) H-Class from Waters Corp. (Milford, MA, USA) coupled with an Evaporative Light Scattering Detector (ELSD) and controlled by the chromatographic software Empower 3 v.3.8.0 from Waters Corp. (Milford, MA, USA) was used to acquire and process method development and validation analytical data. The oxidation stability tester RapidOxy 100 from Anton Paar GmbH (Graz, Austria) was used for oxidative degradation insights. The DoEs were performed with the Acquity HSS T3 (2.1 × 100 mm; 1.8 µm) and UPLC BEH C18 (2.1 × 100 mm; 1.8 µm) columns from Waters Corp. (Milford, MA, USA), and the Triart C18 (2.1 × 100 mm; 1.9 µm) and Triart C18 ExRS (2.1 × 100 mm; 1.9 µm) columns provided by YMC Europe GmbH (Dinslaken, Germany). The DoEs conceptualization and statistical analysis were performed on Design Expert v.13 (Stat-Ease Inc., Minneapolis, MN, USA) and SPSS v.29. (IBM, Armonk, NY, USA). The regression analysis was performed on OriginPro 2023b v.10.0.5.157 (OriginLab Corp., Northampton, MA, USA).

### 3.4. Nanoemulsion Matrix Preparation

A nanoemulsion prototype was internally formulated within the same project to evaluate the validity of the developed analytical methodology. For the preparation of the NEs, the IJM Nanoscaler (Knauer, Wissenschaftliche Geräte GmbH, Berlin, Germany) was employed. It comprised five impingement jet mixers (i.e., IJM 1–5) differing in the inner diameters of the mixers, three pumps (i.e., pump 1–3), and two valves. The organic phase consisted of the liquid lipid Labrafac dissolved in Ethanol absolute. A mixture of the non-ionic stabilizer Tween^®^ 80 and purified water formed the aqueous phase in the stabilizer pump. Mixing of the two phases was performed in a 1:1 (*v*/*v*) ratio using the IJM 3 and a total flow rate of 36.26 mL/min. Subsequently, the formed NEs were further diluted 1:1 (*v*/*v*) with purified water to reach a final concentration of 2 mg/mL of Labrafac and tested.

### 3.5. Analytical Method Validation

#### 3.5.1. Specificity

The specificity of an analytical method is its ability to unequivocally obtain a signal free of any interferences brought by other components, such as different active ingredients, degradation products, or matrix species [28,48]. Unlike photodiode array detectors (PDA) with peak purity functionality, ELSD detectors lack the capability of identifying co-eluting species at the same retention time. Therefore, specificity was assessed by comparing signals from standard injections with those from mobile phase, blank, and matrix formulation component injections.

#### 3.5.2. Forced Degradation Studies

A forced degradation study was performed to provide insight into whether the peak ratios would change, or additional species would appear in the chromatogram. Approximately 30 mg of Labrafac were weighed into 10 mL flasks, and filled up to volume with 2-propanol, obtaining a solution of 3 mg/mL concentration. The solutions were prepared in triplicate, and they were exposed to the following conditions respectively:

- Acid hydrolysis: exposure to 1.0 mL of 6N Hydrochloric Acid solution for 5 h;
- Basic hydrolysis: addition of 100 µL of 6N Sodium Hydroxide solution to 1.0 mL of the reference solution and immediate injection;
- Oxidation: exposure to 1.0 mL of 30% *w*/*v* H_2_O_2_ solution for 5 h;
- Thermolysis: exposure to heat (100 °C) in a dedicated oven for 4 h.

Except for the basic hydrolysis solutions, the samples were filled up with the diluent (2-propanol) and subsequently injected. Additionally, to complement the oxidation data, 5 mL of Labrafac was exposed to a temperature of 200 °C and a relative pressure of 700 KPa in the Rapidoxy 100. The stop criteria were set to a 5% pressure drop. At the end of this stress, the sample was diluted in 2-propanol and analyzed.

#### 3.5.3. Response, LOD, and LOQ

One of the main issues regarding ELSD methodologies is that a linear fit cannot be applied, and Labrafac measurements follow a cubic response, with a 3rd order fit [49]. Nine solutions at concentrations between 1.0 and 5.0 mg/mL were prepared by dilution from three independent stock solutions, by dissolving Labrafac in the diluent [36]. LOD and LOQ were calculated using the signal-to-noise ratio approach, which is relevant for analytical procedures that show baseline noise [28]. The signal-to-noise ratio is determined by comparing measured signals from samples with known low analyte concentrations with those of blank injections. A signal-to-noise ratio of 3:1 was considered acceptable for estimating LOD, and for LOQ, a ratio of at least 10:1 was acceptable [28].

#### 3.5.4. Accuracy

The accuracy of an analytical method indicates the degree of agreement between the expected value or the reference value and the obtained value in the results [28]. Solutions of Labrafac at three concentration levels of 70, 100, and 130% of the declared content/labeled claim were prepared by weighing (*n* = 3) and dissolving in 2-propanol, then further analyzed.

#### 3.5.5. Precision (Repeatability and Intermediate Precision)

The precision of an analytical method expresses the closeness of agreement (degree of scatter) between a series of measurements obtained from multiple sampling of the same homogeneous sample under the prescribed conditions. Repeatability expresses the precision under the same operating conditions over a short time interval [47]. Repeatability is also termed intra-assay precision [28]. The repeatability was investigated by analyzing six independent determinations of Labrafac (*n* = 6). The intermediate precision expresses within-laboratory variations, and it was determined by analyzing an identically prepared set of samples from a different analyst on a different day (*n* = 6). The degree of difference was assessed via the Student’s *t*-test [50].

#### 3.5.6. Robustness

The robustness of an analytical methodology measures its capacity to meet expected performance criteria even when small but intended changes in the method parameters are applied [28,36]. Hence, it offers insight into the method’s reliability in routine operation. This study considers the well-recognized issues of the reproducibility of results obtained with the ELSD [51]. A response surface D-optimal design was conducted, investigating drift tube temperature, nebulizer gas rate, and gas pressure. The data were analyzed using a Type III ANOVA.

## 4. Conclusions

The developed reversed-phase UPLC-ELSD method is suitable for analyzing MCTs in bulk material and NEs prototype formulation. Following AQbD principles and supported by statistical evaluation, the method was validated according to ICH Q2 (R2). Our results illustrate a case study of a systematic approach to method development using AQbD in excipient quantification. This enhanced approach offers enhanced knowledge, reducing the regulatory and overall laboratory costs that might come with traditionally developed analytical methods [52]. The methodology has demonstrated accuracy, precision, selectivity, and robustness, with results fitting the typical non-linear cubic model observed in ELS detectors [53].

## Figures and Tables

**Table 1:** Analytical Target Profile for the determination of Labrafac.

ATP Element	Target	Requirement Reference
Number of Peaks	=4	-
Peak Tailing	<2	[28,36]
Peak Height	Above LOQ	[28,36]
Capacity factor (k’)	>2	[28,36]
Resolution	>2	[28,36]

**Table 2:** Factors and levels of the design of experiments for the analytical method development.

Analytical Column	Organic Modifier (%)	Aqueous Phase (%)	Formic Acid (%)	Sample Diluent	Flow (mL/min)	Injection Volume (μL)	Column Temperature (°C)
(A) (B) (C) (D)	Acetonitrile Methanol	0–4	0–0.5	Acetonitrile Methanol Acetone 2-Propanol	0.3–0.7	1–5	25–38

Notes: Column letters corresponding to (A) Acquity HSS T3 (2.1 × 100 mm; 1.8 µm), (B) Acquity UPLC BEH C18 (2.1 × 100 mm; 1.8 µm), (C) YMC Triart C18 (2.1 × 100 mm; 1.9 µm), (D) YMC Triart C18 ExRS (2.1 × 100 mm; 1.9 µm).

**Table 3:** Analytical method conditions utilized for validation.

Validation Conditions	
Flow Rate	0.41 mL/min
Injection Volume	2.8 µL
Formic Acid Ratio	0.4%
Mobile Phase	Water/Methanol (2.5 : 97.5)
Column	Acquity HSS T3
Column Temperature	33 °C
Sample Diluent	2-Propanol

**Table 4:** Forced degradation studies results for the recovery of Labrafac.

Stress Condition	Recovery (%)	Average (%)	SD
Acidic hydrolysis	88.1 88.3 86.2	87.5	1.3
Basic hydrolysis	10.2 9.8 9.6	9.9	0.3
Oxidation	100.6 96.1 99.4	98.7	2.3
Thermolysis	98.4 98.6 98.4	98.4	0.1

**Table 5:** LOD and LOQ values of Labrafac components.

Component	LOD (µg/mL)	LOQ (µg/mL)
Tricaprylin	1.48	4.94
1,2-caprate-3-caprylate	2.29	7.64
1,2-caprylate-3-caprate	4.14	13.81
Tricaprin	168.91	563.06

**Table 6:** Accuracy results from Labrafac’s method validation.

Percent of Target (%)	Recovery (%)	Average (%)	RSD (%)
70%	98.4 100.2 100.9	99.8	1.3
100%	99.5 99.3 99.0	99.3	0.2
130%	98.8 100.6 102.1	100.5	1.6

**Table 7:** Precision and Student *t*-test results for Labrafac’s method validation.

Parameter	Analyst I	Analyst II
Mean	97.32	99.65
SD	2.25	0.31
RSD	2.30	0.30
SEM	0.03	0.04
n	6	6
*p*-value	0.065	

## Data Availability

All data and materials are present in the manuscript and Supplementary Materials.

## Supplementary Materials

The following supporting information can be downloaded at: https://www.mdpi.com/article/10.3390/molecules30030486/s1, Table S1. Chromatographic DoE results evaluation. (1) Tricaprylin. (2) 1,2-caprate-3-caprylate. (3) 1,2-caprylate-3-caprate. (4) Tricaprin; Table S2. Robustness results for Tricaprylin, K prime correlation; Table S3. Robustness results for Tricaprylin, height correlation; Table S4. Robustness results for Tricaprylin, tailing correlation; Table S5. Robustness results for 1,2-caprate-3-caprylate, resolution correlation; Table S6. Robustness results for 1,2-caprate-3-caprylate, K prime correlation; Table S7. Robustness results for 1,2-caprate-3-caprylate, height correlation; Table S8. Robustness results for 1,2-caprate-3-caprylate, tailing correlation; Table S9. Robustness results for 1,2-caprylate-3-caprate, resolution correlation; Table S10. Robustness results for 1,2-caprylate-3-caprate, height correlation; Table S11. Robustness results for 1,2-caprylate-3-caprate, K prime correlation; Table S12. Robustness results for 1,2-caprylate-3-caprate, tailing correlation; Table S13. Robustness results for Tricaprin, resolution correlation; Table S14. Robustness results for Tricaprin, height correlation; Table S15. Robustness results for Tricaprin, K prime correlation; Table S16. Robustness results for Tricaprin, Tailing correlation.

## Abbreviations

API	Active Pharmaceutical Ingredient
AQbD	Analytical Quality by Design
ATP	Analytical Target Profile
CMeAS	Critical Method Attributes
CMePS	Critical Method Parameters
DoE	Design of Experiments
ELSD	Evaporative Light Scattering Detection
GC	Gas Chromatography
HPLC	High-Performance Liquid Chromatography
HLB	Hydrophilic-lipophilic Balance
ICH	International Council of Harmonization
LOD	Limit of Detection
LOQ	Limit of Quantification
MCTs	Medium Chain Triglycerides
MODR	Method Operable Design Region
NE	Nanoemulsion
PDA	Photo Diode Array
QbD	Quality by Design
QRM	Quality Risk Management
RSD	Relative Standard Deviation
RT	Retention Time
RP	Reversed-phase
USP	United States Pharmacopoeia
UPLC	Ultra High-Performance Liquid Chromatography
UV	Ultraviolet
