# Supplementary material for: AQbD-Based UPLC-ELSD Method for Quantifying Medium Chain Triglycerides in Labrafac™ WL 1349 for Nanoemulsion Applications
Source: Molecules. 2025 Jan 22;30(3):486. doi: 10.3390/molecules30030486 (PMC11820448; doi:10.3390/molecules30030486)
Supplement: Supplementary file 1 [file molecules-30-00486-s001.zip › molecules-3343413-supplementary.pdf]

molecules

MDPI

Article

AQbD-Based UPLC-ELSD Method for Quantifying Medium Chain Triglycerides in Labrafac™ WL 1349 for Nanoemulsion Applications

Alessio Gaggero [1], Viktoria Marko [1], Dalibor Jeremic [2], Carolin Tetyczka [1], Philippe Caisse [3] and Jesús Alberto Afonso Urich [1,4,*]

[1] Research Center Pharmaceutical Engineering GmbH, 8010 Graz, Austria; alessio.gaggero@rcpe.at (A.G.); viktoria.marko@rcpe.at (V.M.); carolin.tetyczka@rcpe.at (C.T.)
[2] Department of Health Studies—Biomedical Science, FH JOANNEUM, 8020 Graz, Austria; dalibor.jeremic@fh-joanneum.at
[3] Gattefossé SAS, 69804 Saint-Priest, France; pcaisse@gattefosse.com
[4] Institute of Process and Particle Engineering, Graz University of Technology, 8010 Graz, Austria
* Correspondence: afonsourich@student.tugraz.at or jesus.afonso@rcpe.at; Tel.: +43-316-873-30988

Table S1. Chromatographic DoE results evaluation. (1) Tricaprylin. (2) 1,2-caprate-3-caprylate. (3) 1,2-caprylate-3-caprate. (4) Tricaprin.

Chromatographic DoE Evaluation							
**Solution 1 of 100 Response**	**Predicted Mean**	**Std Dev**	**n**	**SE Pred**	**95% PI low**	**Data Mean**	**95% PI high**
**K prime (1)**	2.71044	0.777196	6	N/A	2.0391	2.4	3.3291
**Height (1)**	1.47825E+06	263335	6	128161	1.22371E+06	1.91214E+06	1.73279E+06
**Tailing (1)**	1.25336	0.109543	6	N/A	1.11181	1.2	1.38108
**Resolution (2)**	5.44306	1.11716	6	N/A	4.44834	5.39939	6.41202
**K prime (2)**	4.02276	1.18739	6	N/A	3.0004	3.5	4.96123
**Height (2)**	1.59498E+06	290058	6	127359	1.34236E+06	1.91253E+06	1.84759E+06
**Tailing (2)**	1.37087	0.35705	6	N/A	1.04915	1.28571	1.65248
**Resolution (3)**	6.86837	1.26698	6	0.558607	5.75924	7.2	7.9775
**K prime (3)**	5.75815	1.65939	6	N/A	4.24986	5.11647	7.11923
**Height (3)**	843231	350038	6	N/A	532991	1.32887E+06	1.13547E+06
**Tailing (3)**	1.19441	0.0915907	6	N/A	1.08406	1.2	1.2948
**Resolution (4)**	9.4186	1.95305	6	N/A	7.68369	10.8317	11.0693
**K prime (4)**	8.61785	3.00025	6	N/A	5.87177	7.63312	11.0838
**Height (4)**	34158.7	13351.9	6	N/A	21881.6	65326.5	45233.4
**Tailing (4)**	1.02965	0.176655	6	N/A	0.876305	0.968157	1.18521

Table S2. Robustness results for Tricaprylin, K prime correlation.

Dependent Variable: K prime Tricaprylin					
**Source**	**Type III Sum of Squares**	**df**	**Mean Square**	**F**	**Significance**
**Corrected Model**	0.033[1]	6	0.006	0.589	0.736
**Intercept**	114.266	1	114.266	12056.707	<0.001
**Drift Tube Temperature**	0.017	2	0.009	0.916	0.412
**Nebulizer Power**	0.003	2	0.002	0.175	0.841
**Gas Pressure**	0.012	2	0.006	0.634	0.538
**Error**	0.265	28	0.009		
**Total**	191.010	35			
**Corrected Total**	0.299	34			

[1]R Squared = 0.112 (Adjusted R Squared = -0.078).

 https://doi.org/10.3390/molecules30030486

Table S3. Robustness results for Tricaprylin, height correlation.

Dependent Variable: Height Tricaprylin					
**Source**	**Type III Sum of Squares**	**df**	**Mean Square**	**F**	**Significance**
**Corrected Model**	257606868825.801[1]	6	42934478137.634	0.904	0.506
**Intercept**	5232360501817.071	1	5232360501817.071	110.111	<0.001
**Drift Tube Temperature**	43076688505.197	2	21538344252.599	0.453	0.640
**Nebulizer Power**	104399287492.296	2	52199643746.148	1.099	0.347
**Gas Pressure**	73763967076.222	2	36881983538.111	0.776	0.470
**Error**	1330527676208.884	28	47518845578.889		
**Total**	9826247407869.000	35			
**Corrected Total**	1588134545034.686	34			

[1]R Squared = 0.162 (Adjusted R Squared = -0.017).

Table S4. Robustness results for Tricaprylin, tailing correlation.

Dependent Variable: Tailing Tricaprylin					
**Source**	**Type III Sum of Squares**	**df**	**Mean Square**	**F**	**Significance**
**Corrected Model**	0.015[1]	6	0.002	1.999	0.100
**Intercept**	28.881	1	28.881	23233.700	<0.001
**Drift Tube Temperature**	0.012	2	0.006	4.919	0.015
**Nebulizer Power**	0.004	2	0.002	1.538	0.232
**Gas Pressure**	0.003	2	0.002	1.371	0.270
**Error**	0.035	28	0.001		
**Total**	49.020	35			
**Corrected Total**	0.050	34			

[1]R Squared = 0.300 (Adjusted R Squared = 0.150).

Table S5. Robustness results for 1,2-caprate-3-caprylate, resolution correlation.

Dependent Variable: Resolution 1,2-caprate-3-caprylate					
**Source**	**Type III Sum of Squares**	**df**	**Mean Square**	**F**	**Significance**
**Corrected Model**	0.578[1]	6	0.096	1.562	0.195
**Intercept**	695.362	1	695.362	11271.576	<0.001
**Drift Tube Temperature**	0.088	2	0.044	0.710	0.500
**Nebulizer Power**	0.041	2	0.021	0.336	0.717
**Gas Pressure**	0.401	2	0.201	3.254	0.054
**Error**	1.727	28	0.062		
**Total**	1156.620	35			
**Corrected Total**	2.306	34			

[1]R Squared = 0.251 (Adjusted R Squared = 0.090)

Table S6. Robustness results for 1,2-caprate-3-caprylate, K prime correlation.

Dependent Variable: K prime 1,2-caprate-3-caprylate					
**Source**	**Type III Sum of Squares**	**df**	**Mean Square**	**F**	**Significance**
**Corrected Model**	0.089[1]	6	0.015	0.864	0.533
**Intercept**	244.123	1	244.123	14220.012	<0.001
**Drift Tube Temperature**	0.037	2	0.018	1.074	0.355
**Nebulizer Power**	0.012	2	0.006	0.337	0.716
**Gas Pressure**	0.047	2	0.024	1.371	0.270
**Error**	0.481	28	0.017		
**Total**	409.260	35			
**Corrected Total**	0.570	34			

[1]R Squared = 0.156 (Adjusted R Squared = -0.025).

Table S7. Robustness results for 1,2-caprate-3-caprylate, height correlation.

Dependent Variable: Height 1,2-caprate-3-caprylate					
**Source**	**Type III Sum of Squares**	**df**	**Mean Square**	**F**	**Significance**
**Corrected Model**	360428126521.016[1]	6	60071354420.169	0.995	0.448
**Intercept**	13956237398580.740	1	13956237398580.740	231.280	<0.001
**Drift Tube Temperature**	136695564890.440	2	68347782445.220	1.133	0.336
**Nebulizer Power**	87801661766.209	2	43900830883.105	0.728	0.492
**Gas Pressure**	116506180505.659	2	58253090252.829	0.965	0.393
**Error**	1689618804579.384	28	60343528734.978		
**Total**	24779769843814.000	35			
**Corrected Total**	2050046931100.400	34			

[1]R Squared = 0.176 (Adjusted R Squared = -0.001).

Table S8. Robustness results for 1,2-caprate-3-caprylate, tailing correlation.

Dependent Variable: Tailing 1,2-caprate-3-caprylate					
**Source**	**Type III Sum of Squares**	**df**	**Mean Square**	**F**	**Significance**
**Corrected Model**	0.009[1]	6	0.001	0.863	0.534
**Intercept**	33.533	1	33.533	19866.852	<0.001
**Drift Tube Temperature**	0.006	2	0.003	1.728	0.196
**Nebulizer Power**	0.001	2	0.001	0.411	0.667
**Gas Pressure**	0.001	2	0.000	0.220	0.804
**Error**	0.047	28	0.002		
**Total**	57.400	35			
**Corrected Total**	0.056	34			

[1]R Squared = 0.156 (Adjusted R Squared = -0.025).

Table S9. Robustness results for 1,2-caprylate-3-caprate, resolution correlation.

Dependent Variable: Resolution 1,2-caprylate-3-caprate					
**Source**	**Type III Sum of Squares**	**df**	**Mean Square**	**F**	**Significance**
**Corrected Model**	1.126[1]	6	0.188	0.664	0.679
**Intercept**	986.936	1	986.936	3491.029	<0.001
**Drift Tube Temperature**	0.290	2	0.145	0.513	0.604
**Nebulizer Power**	0.041	2	0.021	0.073	0.930
**Gas Pressure**	0.681	2	0.340	1.204	0.315
**Error**	7.916	28	0.283		
**Total**	1638.340	35			
**Corrected Total**	9.042	34			

[1]R Squared = 0.125 (Adjusted R Squared = -0.063).

Table S10. Robustness results for 1,2-caprylate-3-caprate, height correlation.

Dependent Variable: Height 1,2-caprylate-3-caprate					
**Source**	**Type III Sum of Squares**	**df**	**Mean Square**	**F**	**Significance**
**Corrected Model**	101487041315.400[1]	6	16914506885.900	1.066	0.406
**Intercept**	1515690641980.627	1	1515690641980.627	95.557	<0.001
**Drift Tube Temperature**	4800113063.841	2	2400056531.920	0.151	0.860
**Nebulizer Power**	12165030529.844	2	6082515264.922	0.383	0.685
**Gas Pressure**	67123798811.942	2	33561899405.971	2.116	0.139
**Error**	444125988701.171	28	15861642453.613		
**Total**	2904821510728.000	35			
**Corrected Total**	545613030016.571	34			

[1]R Squared = 0.186 (Adjusted R Squared = 0.012).

Table S11. Robustness results for 1,2-caprylate-3-caprate, K prime correlation.

Dependent Variable: K prime 1,2-caprylate-3-caprate					
**Source**	**Type III Sum of Squares**	**df**	**Mean Square**	**F**	**Significance**
**Corrected Model**	0.151[1]	6	0.025	00.523	0.786
**Intercept**	522.653	1	522.653	10858.771	<0.001
**Drift Tube Temperature**	0.067	2	0.033	0.691	0.509
**Nebulizer Power**	0.018	2	0.009	0.190	0.828
**Gas Pressure**	0.068	2	0.034	0.705	0.503
**Error**	1.348	28	00.048		
**Total**	874.500	35			
**Corrected Total**	1.499	34			

[1]R Squared = 0.101 (Adjusted R Squared = -0.092).

Table S12. Robustness results for 1,2-caprylate-3-caprate, tailing correlation.

Dependent Variable: Tailing 1,2-caprylate-3-caprate					
**Source**	**Type III Sum of Squares**	**df**	**Mean Square**	**F**	**Significance**
**Corrected Model**	0.013[1]	6	0.002	0.520	0.788
**Intercept**	36.421	1	36.421	8624.573	<0.001
**Drift Tube Temperature**	0.003	2	0.001	0.303	0.741
**Nebulizer Power**	0.009	2	0.005	1.101	0.346
**Gas Pressure**	0.001	2	0.001	0.165	0.849
**Error**	0.118	28	0.004		
**Total**	61.910	35			
**Corrected Total**	0.131	34			

[1]R Squared = 0.100 (Adjusted R Squared = -0.092).

Table S13. Robustness results for Tricaprin, resolution correlation.

Dependent Variable: Resolution Tricaprin					
**Source**	**Type III Sum of Squares**	**df**	**Mean Square**	**F**	**Significance**
**Corrected Model**	1.341[1]	6	0.223	0.211	0.970
**Intercept**	1552.478	1	1552.478	1467.657	<0.001
**Drift Tube Temperature**	0.179	2	0.089	0.084	0.919
**Nebulizer Power**	0.774	2	0.387	0.366	0.697
**Gas Pressure**	0.028	2	0.014	0.013	0.987
**Error**	29.618	28	1.058		
**Total**	2580.150	35			
**Corrected Total**	30.959	34			

[1]R Squared = 0.043 (Adjusted R Squared = -0.162).

Table S14. Robustness results for Tricaprin, height correlation.

Dependent Variable: Height Tricaprin					
**Source**	**Type III Sum of Squares**	**df**	**Mean Square**	**F**	**Significance**
**Corrected Model**	318118337.745[1]	6	53019722.957	1.178	0.346
**Intercept**	3489657082.481	1	3489657082.481	77.559	<0.001
**Drift Tube Temperature**	62689792.368	2	31344896.184	0.697	0.507
**Nebulizer Power**	110220782.058	2	55110391.029	1.225	0.309
**Gas Pressure**	108814084.973	2	54407042.487	1.209	0.314
**Error**	1259828180.427	28	44993863.587		
**Total**	7151998600.000	35			
**Corrected Total**	1577946518.171	34			

[1]R Squared = 0.202 (Adjusted R Squared = 0.031).

Table S15. Robustness results for Tricaprin, K prime correlation.

Dependent Variable: K prime Tricaprin					
**Source**	**Type III Sum of Squares**	**df**	**Mean Square**	**F**	**Significance**
**Corrected Model**	0.450[1]	6	0.075	0.649	0.690
**Intercept**	1157.722	1	1157.722	10029.645	<0.001
**Drift Tube Temperature**	0.190	2	0.095	0.823	0.449
**Nebulizer Power**	0.065	2	0.033	0.283	0.756
**Gas Pressure**	0.193	2	0.097	0.837	0.444
**Error**	3.232	28	0.115		
**Total**	1932.140	35			
**Corrected Total**	3.682	34			

[1]R Squared = 0.122 (Adjusted R Squared = -0.066).

Table S16. Robustness results for Tricaprin, Tailing correlation.

Dependent Variable: Tailing Tricaprin					
**Source**	**Type III Sum of Squares**	**df**	**Mean Square**	**F**	**Significance**
**Corrected Model**	0.062[1]	6	0.010	0.198	0.975
**Intercept**	33.843	1	33.843	654.341	<0.001
**Drift Tube Temperature**	0.005	2	0.002	0.048	0.953
**Nebulizer Power**	0.037	2	0.019	0.358	0.702
**Gas Pressure**	0.018	2	0.009	0.169	0.845
**Error**	1.448	28	0.052		
**Total**	60.920	35			
**Corrected Total**	1.510	34			

[1]R Squared = 0.041 (Adjusted R Squared = -0.165).
